# Variable co-adaptation: exploring the emergence of teacher-student interaction patterns in award-winning EFL classes through the CDST lens

**DOI:** 10.3389/fpsyg.2026.1783591

**Published:** 2026-03-09

**Authors:** Binfeng Chen, Weining Zhang, Yihan Xie

**Affiliations:** College of Zhicheng, Fuzhou University, Fuzhou, China

**Keywords:** CDST, co-adaptation, emergence, teacher-student interaction, teaching contest, variability

## Abstract

**Introduction:**

Teacher-student interaction (TSI) is core to effective foreign language teaching (FLT), yet its linguistic-cognitive dynamics are under-explored via Complex Dynamic Systems Theory (CDST). SFLEP Cup teaching contests form natural complex dynamic systems with fixed students and rotating teachers, offering a unique context to explore TSI emergence.

**Methods:**

We analyzed 20-minute demo lessons of 10 award-winning EFL teachers, extracting 315 teacher-student question-answer pairs. State Space Grids (SSGs) visualized TSI dynamics; a 4-point dual coding framework was applied to video data. Spearman's correlation analysis (α = 0.05) examined bivariate associations between key TSI variables.

**Results:**

Individual teachers' adaptive variability converged into a dominant global TSI attractor state: simple elicitation questions paired with concise student answers. Linguistic TSI coupling (mean ρ = 0.771) was far stronger than cognitive coupling (mean ρ = 0.377). Effective TSI elicitation correlated positively with contest rankings, and variability/co-adaptation drove global pattern emergence from heterogeneous local teacher dynamics.

**Discussion:**

The low-cognitive-demand TSI pattern is shaped by contest evaluation priorities and performative teaching characteristics. Teachers designed for higher-order thinking, but student cognitive output failed to match, revealing linguistic-cognitive misalignment. Variability and co-adaptation are core mechanisms for global pattern emergence from local dynamics. We call for integrating cognitively challenging interactions into routine FLT and refining contest criteria to prioritize cognitive activation over surface-level interaction fluency, extending CDST applications in L2 classroom research.

## 1 Introduction

The escalating integration of artificial intelligence in education has reshaped the classroom landscape and altered the nature of teacher-student interaction (TSI) (Tai and Chen, 2024). This shift poses challenges for foreign language teaching (FLT), as authentic, in-person TSI is not merely beneficial but indispensable to effective language acquisition (Mercer and Dörnyei, 2020). Addressing these challenges, language teaching contests, a microcosm of outstanding teaching and a catalyst for teacher professional development (Li and Li, 2013; Tang and Shao, 2014; Huang et al., 2024), exemplified by the SFLEP Cup National Foreign Language Teaching Contest (SNFLTC), have sought to differentiate themselves from general teaching contests by recruiting on-site college students and requiring contestants to engage in authentic, real-time interaction. All this highlights the urgent need to revisit and unpack the intricate dynamics of TSI in contemporary language classrooms (Larsen-Freeman and Cameron, 2008; Shu et al., 2023).

Notably, such dynamics have been increasingly explored through the lens of Complex Dynamic Systems Theory (CDST) (Larsen-Freeman, 2022). SNFLTC is especially well-suited to CDST inquiry as students remain relatively consistent across sessions while teachers rotate, a naturally dynamic system with built-in variability. While CDST rejects oversimplification of complex phenomena, it does not preclude the identification of meaningful patterns (van Dijk et al., 2024). A core tenet of CDST is reconciling global regularities with local variability, complexity, and context-specificity in the processes (Thelen and Smith, 1994). In this regard, CDST not only accounts for idiosyncratic trajectories in individual cases but also illuminates overarching orders and patterns beyond isolated features (Li and Zheng, 2025).

Building on CDST's emphasis on adaptive, non-linear processes, Smit et al. (2022) examined teacher-student co-adaptation via question-answer sequences in English-as-a-Foreign-Language (EFL) contexts. Despite this progress, existing research often overlooks a defining duality of FLT: language functions simultaneously as the instructional content and the medium for mastering that content (Gibbons, 2015; Dalton-Puffer et al., 2010). This duality means classroom interaction operates at both linguistic and cognitive dimensions, yet how these two dimensions synergize to drive effective TSI remains under-explored (Peng and Yu, 2025; Huang et al., 2025). While such frameworks as the Questions and Answers in English Language Teaching (QAELT) scheme (Smit et al., 2022) offer valuable insights into interactional structures, their lack of an integrated lens for capturing the linguistic-cognitive interplay constrains their capacity to fully explain the complexity of effective FLT interaction.

Against this backdrop, the present study draws on CDST to investigate the emergence of TSI patterns by analyzing classroom demos from SNFLTC award-winning teachers. Unlike most existing CDST-informed studies that focus on individual teachers' TSI development, this study examines award-winning exemplar teachers as a collective group to illuminate the characteristics of high-quality FLT in China. While the data are derived from national teaching contest demos, the identified interaction patterns hold transferable insights for both contest-oriented and routine classroom settings, offering a scalable framework for enhancing TSI quality across diverse EFL teaching scenarios. Theoretically, the study extends the QAELT framework (Smit et al., 2022) by integrating explicit linguistic and cognitive dimensions, thereby addressing the need for a more comprehensive tool to explicate language-pedagogic exchange (Walsh, 2011; Peng and Yu, 2025). Empirically, the study identifies emerging TSI patterns that can inform evidence-based FLT practice, particularly in AI-integrated classrooms where authentic TSI is increasingly increasingly challenged (Tai and Chen, 2024). In so doing, the study responds to the urgent call for research that bridges theoretical gaps and offers practical guidance for sustaining high-quality TSI in contemporary language education.

## 2 Literature review

### 2.1 TSI as a complex dynamic system

TSI has long been recognized as essential in language acquisition (Hattie and Yates, 2014; Mercer and Dörnyei, 2020), yet its inherent complexity, particularly in prominent contexts like teaching contests, resists reductionist explanations. For instance, Larsen-Freeman (2020) emphasized that learners, as active human agents in situated classroom interactions, reshape the interaction's nature, intensity, and underlying dynamics. Hence, CDST has emerged as a powerful lens to unpack this intricacy, framing TSI as a non-linear, adaptive, and emergent phenomenon rather than a fixed set of prescriptive behaviors (de Bot et al., 2007a; Larsen-Freeman, 2022).

In CDST, local variability and global patterns are equally critical to sustaining systemic dynamics. Variability is conceptualized as differences in the level of a developmental variable, encompassing both intra-individual fluctuations and variations across repeated measurements (van Geert and van Dijk, 2002). Global patterns, by contrast, refer to identifiable and relatively stabilized types, skills, schemas, or achievement configurations that emerge through the process of self-organization within the system (Dörnyei, 2014). This interdependence aligns with CDST's core emphasis on how dynamic interactions between variable local processes foster the emergence of coherent global structures, which in turn shape subsequent developmental trajectories. Rooted in systems thinking, CDST further conceptualizes classroom interaction as a self-organizing process, where patterns emerge through continuous mutual adjustments and feedback loops between participants (Larsen-Freeman and Cameron, 2008). A cornerstone of this framework, “emergence” denotes the manifestation of a new state at a higher level of analysis than its constituent components, accompanied by the emergence of unique properties not present in the system's individual parts (Byrne and Callaghan, 2014; Larsen-Freeman and Cameron, 2008). Over time, these emergent patterns may stabilize into “attractor states,” self-replicating, self-sustaining interactional norms that resist external change (van Dijk et al., 2024). Disrupting such entrenched states requires significant effort, even as the system retains inherent variability that enables flexibility (de Bot et al., 2007a; Hollenstein, 2013). This dynamism allows the system to adapt to contextual stimuli, generating context-sensitive variability that drives evolutionary change (de Bot et al., 2007b; Hiver and Larsen-Freeman, 2020).

CDST-informed research on TSI has yielded some empirical insights. Smit et al. (2022) demonstrated that EFL TSI operates as a complex system, where teacher questions and student responses co-evolve through reciprocal adaptation rather than one-way transmission. Extending this line of inquiry, Huang et al. (2025) and Peng and Yu (2025) applied it to Chinese as a second language classrooms and postgraduate EFL learners' online supervisor interactions, respectively. Together, these studies underscore CDST's utility in explaining the dynamic, context-dependent nature of L2 classroom interaction.

### 2.2 The interplay of linguistic and cognitive dimensions of FLT

The interplay between linguistics and cognition, a cornerstone of cognitive linguistics (Dancygier, 2017; Xu and Taylor, 2021), has been further illuminated by CDST. CDST-informed research reveals that the L2 system is characterized by complex, non-linear interactions across multiple dimensions (Evans and Larsen-Freeman, 2020; Lesonen et al., 2021). This understanding has prompted a shift in language research from a mono-scale to a multi-scale perspective, which emphasizes the nested dynamics across different levels of analysis (Thibault, 2011; de Bot, 2015).

Paralleling this epistemological shift, FLT research has increasingly reconceptualized L2 development as a dual semiotic-cognitive or structural-functional process, dynamically co-constructed through context-embedded social interaction (Ellis, 2019; Gibbons, 2015). Within this integrative framework, the linguistic dimension is no longer narrowly defined by accuracy but is expanded to encompass lexico-grammatical complexity, syntactic sophistication, and pragmatic appropriateness as interconnected constructs (Bednarek, 2009). Concurrently, the cognitive dimension in FLT has prioritized higher-order thinking, with empirical studies confirming that collaborative tasks and meaning co-construction foster deeper cognitive processing than individual activities (Mercer and Dörnyei, 2020).

The synergy between these two dimensions is thus not merely sequential but reciprocal (Pan et al., 2023). This is evidenced by empirical findings which demonstrate that complex reasoning tasks correlate with more sophisticated lexico-grammatical use (Zhao et al., 2024), a phenomenon aligned with Bednarek's (2009) framework linking linguistic choices to cognitive judgments. Consequently, the interplay of structural/linguistic and functional/cognitive processes is not only revealed in controlled experiments but is also fundamentally embedded in the very dynamics of language learning and teaching.

## 3 Research questions

Building on CDST's core tenets of reconciling global regularities with local variability (Thelen and Smith, 1994; Larsen-Freeman, 2022) and addressing the identified gap in integrating linguistic and cognitive dimensions of TSI research (Smit et al., 2022; Peng and Yu, 2025; Huang et al., 2025), this study aims to unpack the interactional dynamics of award-winning EFL teachers. The study is thus guided by the following research questions:

**RQ1**. What are the structural and functional characteristics of the global TSI patterns among award-winning teachers?

**RQ2**. How do these global TSI patterns emerge from the local dynamics of individual teachers?

## 4 Methodology

### 4.1. Data sources and collection

The present study examines the instructional practices of the 10 grand-prize winners of the 15th SNFLTC in 2024. Unlike generic university teaching contests, SNFLTC is explicitly designed for tertiary-level English instructors, spanning English-major programs, EAP/ESP courses, and vocational-college English tracks. The 2024 cycle was open to all English-major teachers nationwide encompassing preliminary, semi-final, and final rounds adjudicated by a panel of discipline experts. The 10 participants analyzed here constitute the entire cohort of national top-prize recipients (Table 1). Although the sample is modest in absolute terms, it represents the population apex of Chinese university English teaching in 2024. Each demo lesson is the product of iterative design, rehearsal, and expert mentoring, thereby functioning as a condensed but authentic instantiation of the teacher's enacted pedagogy.

**Table 1:** Teacher profile.

**Teacher**	**Gender**	Score^*^ (Total: 100)	**Ranking**
A	Female	91.49	1
B	Female	89.18	6
C	Female	88.53	10
D	Female	89.59	4
E	Female	89.03	7
F	Male	90.06	3
G	Male	88.83	8
H	Female	90.45	2
I	Female	89.24	5
J	Male	88.54	9

^*^To ensure accuracy, the scores and rankings employed in this study are exclusively those of the contestants' teaching demonstrations officially released by the organizing committee.

The dataset analyzed in this study was from 10 official We Chat articles released by the authorized account “SELEP-HE.” Consent was obtained from the contest organizing committee, which holds the copyright for all textual and audiovisual materials. The primary analytical units consisted of 10 award-winning video recordings of finalists, which were randomly labeled A through J to mitigate potential bias associated with their existing rankings. Each video was approximately 20 mins in length, including 3-mins teaching-design rationale and 17-mins in-class teaching demonstration based on the designated teaching materials. While the teaching demonstrations constitute the primary data source, the teaching-design introductions served as complementary evidence to contextualize and interpret the pedagogical practices under investigation.

All video recordings were transcribed verbatim into English with the aid of Tingwu (tingwu.aliyun.com), a transcription platform specializing in audio transcription. The first and second authors independently reviewed each transcript against the original audio; discrepancies were resolved through re-listening and consensus. This two-step procedure can minimize transcription-induced artifact and maximize the reliability of subsequent analysis.

### 4.2 Coding procedure

Although the present study adopted the QAELT taxonomy proposed by Smit et al. (2022) and refined by Huang et al. (2025), the original matrix was found to conflate linguistic form with cognitive demand. As L2 development is simultaneously a semiotic and a cognitive accomplishment through social-interaction in a specific context over time (Ellis, 2019; Gibbons, 2015; Seuren, 2001), we disambiguated the two strata by introducing a bifurcated analytic framework that operationalizes both linguistic level and cognitive level. It is important to clarify that the two levels coded are derived from observable discourse features rather than direct measurements of learners' internal affective states or unobservable cognitive processes. This aligns with Smit et al. (2022) whose coding focuses on manifest patterns as proxies for underlying processes, avoiding over-interpretation of internal states, an inherent limitation of non-intrusive classroom observation (Mercer and Dörnyei, 2020).

Coding proceeded in two iterative stages: event demarcation and level assignment. In Stage 1, consistent with Smit et al. (2022), an “event” was defined as the minimal adjacency pair comprising a teacher question and the immediately ensuing student response (Hiver and Al-Hoorie, 2016). Each transcribed lesson was imported into Excel; start-/end-times were logged for every event. Re-starts, reformulations or follow-up probes initiated by the teacher were treated as discrete events. Procedural or phatic utterances that did not solicit curriculum-relevant content (e.g., “How's your day?”, “Right?”) were excluded, mirroring the filtering protocol advanced by Huang et al. (2025).

In Stage 2, every retained question-response pair was double-coded on two 4-point ordinal scales (0 = N/A, 1 = low, 2 = medium, 3 = high) reflecting linguistic complexity and cognitive complexity (see Table 2). The descriptors integrate Anderson and Krathwohl's (2001) revised Bloom taxonomy and Gibbons' (2015) scaffolding framework, ensuring compatibility with previous QAELT applications while foregrounding the linguistic-cognitive distinction central to the present study.

**Table 2:** Coding scheme of this study (adapted from Smit et al., 2022).

**Dimension**		Teacher's question (Tq)		Student's answer (Sa)	
		**Description**	**Code**	**Description**	**Code**
Linguistic/Structural Dimension	N/A	The teacher poses a statement or question but does not elicit a Sa (e.g., no pause provided for the student to answer).	0	The student does not respond to the Tq, or the Sa is irrelevant to the Tq.	0
	low	Closed questions with pre-defined answers (e.g., yes/no questions, multiple-choice prompts) or questions requiring only sentence completion or short-phrase responses.	1	The student provides a very short answer (1-3 words), or asks for clarification on the task (e.g., “What should I do?”), or reads aloud from the assignment or text.	1
	medium	Semi-open questions, typically descriptive, that prompt basic elaboration beyond short phrases.	2	The student answers the question with information directly derived from lesson content, which demonstrates familiarity with both the language used and the content. Or the student answers the question with adequate information but not in correct grammar.	2
	high	Open questions designed to elicit extended language use, encouraging students to verbalize answers in full, connected sentences.	3	The student provides a complex or lengthy response (more than one sentence) and/or expands on the lesson by introducing new, relevant elements. The grammar is basically right and the structure is complete.	3
Cognitive/Functional Dimension	N/A	It is more a display than an elicitation question and there is no room for student interpretation/thinking.	0	The student does not respond to the Tq, or the Sa is irrelevant to the Tq.	0
	low	Questions designed to check factual knowledge or assess whether the student has understood or retained specific content.	1	The answer reflects the student's ability to recall basic facts or grasp fundamental concepts, without involving higher-order cognitive skills.	1
	medium	This category includes questions that require students to explain ideas or summarize content in their own words.	2	The answer demonstrates the student's intermediate cognitive ability, eg., explaining or summarizing information based on a solid understanding, without reaching higher cognitive levels.	2
	high	Questions aimed at exploring reasons, opinions, predictions, justifications, or meta-cognitive reflections. These are probing questions intended to challenge students' perspectives, focusing on meaning co-construction.	3	The answer showcases the student's high-level cognitive competence, as evidenced by skills such as analysis, evaluation, application, or creation.	3

To ensure coding reliability, three authors engaged in a three-stage consensus-oriented procedure rather than classical inter-rater statistics considering the comparatively small data-set. First, two calibration meetings were held to pilot-code one randomly selected lesson; discrepancies were discussed until agreement was reached and the coding scheme was correspondingly refined. Second, the second and third authors independently coded the entire dataset, with the inter-rater reliability (calculated via percent agreement) reaching over 93%. The first author then collated the two coding sets, identified discrepancies, and organized repeated triangulation sessions. In these sessions, each divergent coding instance was re-examined alongside the original classroom video and transcript until unanimous consensus was reached. This dialogic reconciliation approach increases coder reliability without violating the small-N assumption underlying intensive, multi-analyst research designs (Campbell et al., 2013). Exemplar coding is provided in Table 3 with some necessary explanations.

**Table 3:** Coding examples.

**NO**.	**Tq**	**Linguistic dimension coding**	**Cognitive dimension coding**	**Sa**	**Linguistic dimension coding**	**Cognitive dimension coding**
1	Do you think he's a happy man?	1 (closed question with a simple Y/N response)	2 (evaluative question asked in a simple way)	No.	1 (a simple Y/N response)	1 (The student does not provide further explanation.)
2	Is it just a favor that they did?	0 (The teacher did not leave enough time for students to answer.)	1 (The teacher elicited students to think.)	(No response.)	0	0
3	Can you read it please?	1	0 (Reading did not elicit any thinking.)	He skipped down the hallway. She smiled as white as the sky.	1	0
4	What are other foolish opinions in the text?	2	2	Aristotle could have avoided the mistake of thinking that, um women have fewer teeth.	2	2
5	Is it possible to break gender stereotypes and realize gender equality? If you answer is yes, what can you offer as a language learner? If your answer is no, what are you worried about?	3 (The teacher guided students to answer questions in extended language use.)	3 (critical thinking question)	I think as a language learner we have the responsibility to express ourselves about break(ing) the gender stereotypes or realize the gender equality. We have to express and to communicate these thins to the different language users.	3	3

### 4.3 Data analysis

The coded data were analyzed with GridWare 1.15 (Hollenstein, 2013), a state-space grid (SSG) technique that visualizes complex dynamic systems as two-dimensional scatterplots. A 4 × 4 grid was constructed in which each cell represents a unique configuration of teacher-question level (rows) and student-answer level (columns). The software exports both static indices and dynamic indices of flexibility/rigidity: (1) events: the raw count of question-answer pairs per contestant; (2) cell range: the number of distinct grid cells visited; (3) dispersion coefficient: a bias-corrected measure of variability ranging from 0 (all events in one cell) to 1 (perfectly even distribution) (Hollenstein, 2013); (4) grid-visit entropy: a direct measure of the unpredictability of the trajectory event-to-event sequence (Dishion et al., 2004).

To identify dominant interaction patterns, attractor values were computed for each contestant. The cell with the highest Q-A frequency was designated the attractor state; its count was divided by the contestant's total events. Higher percentages indicate stronger attractor effects. Complementing the grid analyses, bivariate associations were examined in SPSS 31.0 via Spearman's ρ (two-tailed, α = 0.05): (1) ordinal-time Q-A series in two dimensions (between teacher question level and subsequent student response level); (2) lagged Q-A series in two dimensions (between each student response level and the next teacher question level); (3) cross-dimensional coupling (between linguistic level and cognitive level). These analyses illuminate how linguistic and cognitive demands co-evolve and how their coupling relates to instructional quality.

**Figure 1:**
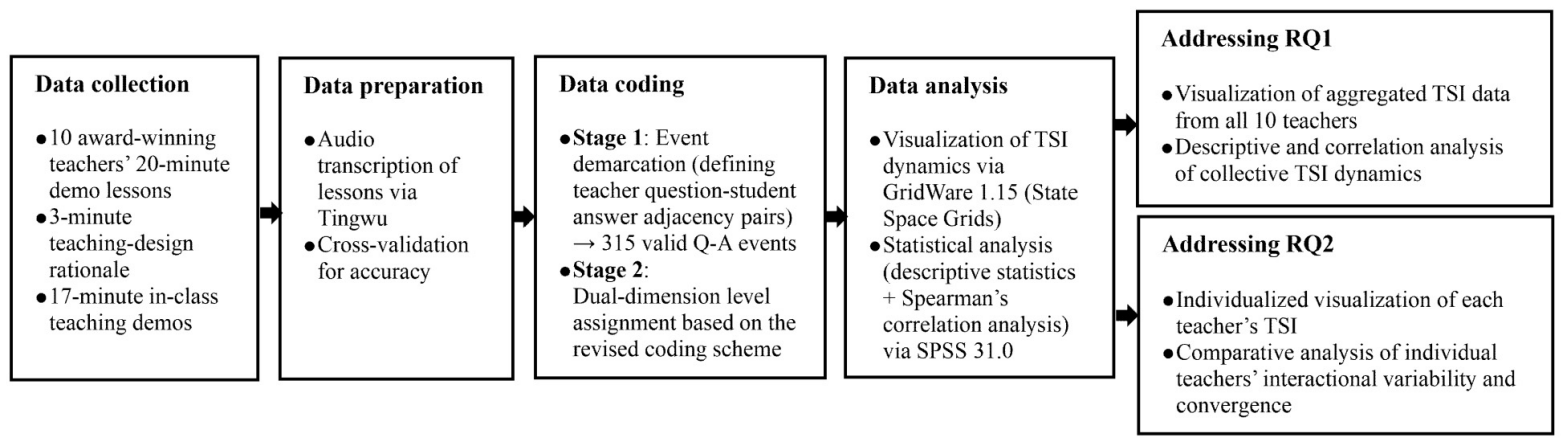
The flowchart of data analysis procedure.

Figure 1 illustrates the workflow of data collection, preparation, coding, and analysis employed to address the study's research questions.

## 5 Result

### 5.1 Trends and patterns of award-winning teachers' Q and A interaction

**Figure 2:**
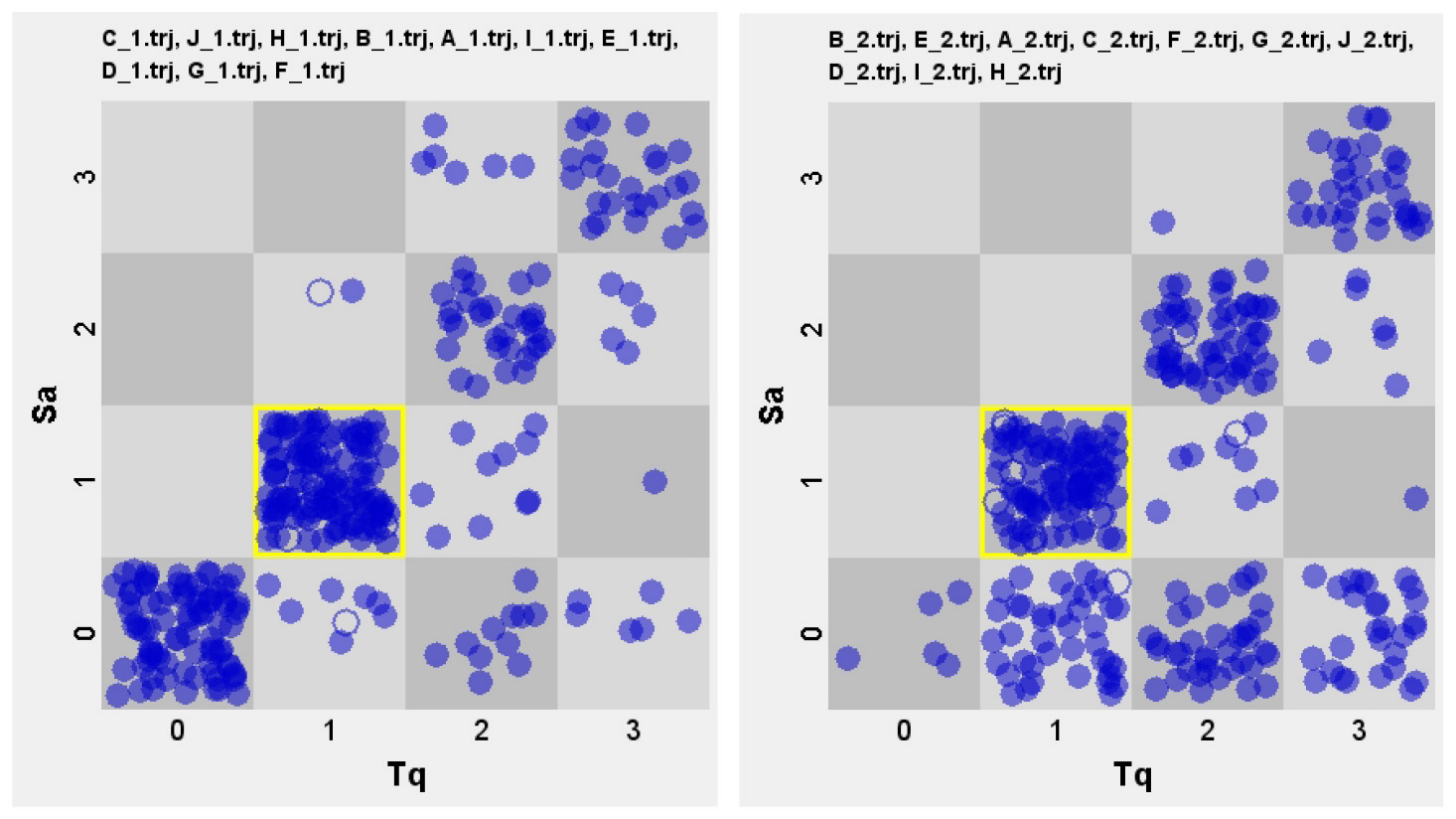
Overview of the structural **(left)** and functional **(right)** TSI pattern of award-winning teachers.

Figure 2 reveals how award-winning teachers orchestrate classroom talk along two complementary tracks. In the structural/linguistic dimension (left), their questions cluster at lower to medium complexity scores (0-1), a level that systematically stretches students' lexical and syntactic resources while remaining within reachable bounds. The attractor state is closed-short where teacher question is closed with a short and simple response, reaching to as high as 124 events out of 315 (attractor value: 39.4%). The next dominant pattern is the 0-0 grid (85 events) where almost no interaction is observed.

Shifting to the functional/cognitive dimension (right), the attractor state is also low-low cognitive grid, reaching 98 out of 315 (attractor value: 31.1%). The second and third dominant pattern is medium-medium (56), indicating a comparatively better cognitive interaction, meaning teachers escalating toward analysis or synthesis, creating a scaffolded ascent of thinking. A noticeable difference is that in this dimension the nodes move somewhat lower-right, meaning that while teachers elicit higher-order thinking, students performance might not reach the intended results. Another thing worth mentioning is the very small number of 0-0 grid (only 5 out of 315), in stark contrast with that in the linguistic dimension, which means that teachers and students actively calibrating cognitive load to the higher order. This emphasis on the development of thinking skills, particularly critical thinking, alongside analytical, research, communicative, and cross-cultural thinking capacities, is also fully reflected in the teaching designs of all 10 teachers, as illustrated in Table 4. Their designs integrate thinking skill cultivation into instructional models, procedures, and objectives, ranging from critical analysis of wisdom and narrative writing to the mastery of rhetorical strategies and the development of value systems oriented toward truth-seeking.

**Table 4:** Excerpts from the teaching design introduction.

**Teacher**	**Excerpts**
A	***Guided by bloom's taxonomy***, I devised the I-STEC teaching model, so my class is integrated skills-focused, student-centered, task-based, e-teaching-added, and cultural-competence- and ***critical-thinking-based*** with more education going through the how teaching process.
B	My teaching philosophy is ***based on Bloom's Taxonomy***. This aligns with my teaching objectives with its clear hierarchical progression and also it gives you very clear clue of what kind of scaffolding steps students are about to climb on. In teaching methodology, I have designed*** Ascending Analysis*** along with blended learning...My teaching procedures have four parts, which I developed into ***read, analyze, create and become***.
C	After that, they think critically and then write a narrative essay by using different narrations.
D	The third goal here is to ***cultivate the students ability to question***, strengthen their understanding of truth, thus developing a correct value system that guides them to pursue truth in their daily lives as the ultimate goal.
E	On the ability level...students shall learn to ***think critically*** of what wisdom is...
F	Students will also need to ***critically think*** about staying foolish or being wiser.
G	...cross culture learning and ***critical thinking*** would be very essential for their future studies and creative shaping.
H	The skills objectives include ***thinking ability***, research ability and communicative competence.
I	I will help our students to learn about the three rhetorical uses, ethos, pathos and logos in persuading other people, and that we will advance to the ability level, which is of course focusing on the ***critical thinking skills***.
J	Second, to cultivate my students ***critical thinking*** and reading ability...

### 5.2 Individual teachers' variation in Q and A interaction

#### 5.2.1 TSI in the linguistic dimension

**Figure 3:**
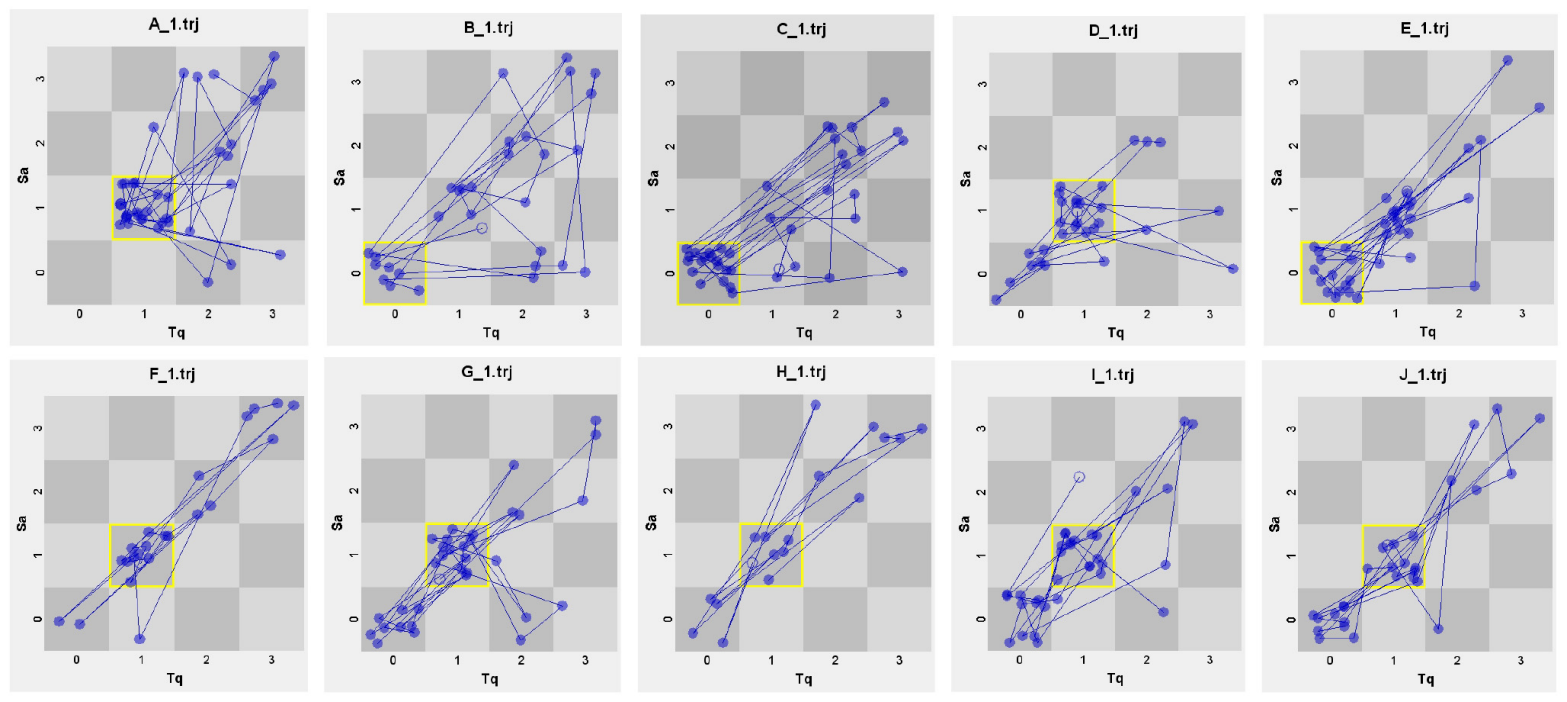
TSI of individual teachers in the linguistic dimension.

Figure 3 reveals the micro-level profiles of the 10 teachers in the linguistic dimension. Teachers B, C and D are clustered around a “non-elicitation” category: most of their moves stop at informing or rhetorical questioning, generating minimal student language. The remaining seven teachers operate mainly in a “closed(question)-short(answer)” pattern with quick, display questions that at least extract a brief verbal response. Strikingly, for E, G, I and J the two regimes are almost level: the “non-elicitation” share sits just one or two percentage points below the “closed-short” share, signaling an unstable, low-elicitation equilibrium. Teacher A stands out as the only participant with “zero” non-elicitation turns. When the interaction data are lined up against the ranking, a clear gradient appears: Teacher A, who won the top prize, shows the lowest incidence of low-elicitation talk; conversely, the three teachers whose profiles are dominated by non-elicitation occupy the bottom ranks.

Table 5 provides a detailed data of each contestant's performance in the linguistic dimension. Noticeably, Teacher A has the highest attractor value (58.97%) and the lowest dispersion coefficient (0.665), indicating the most concentrated and stable pattern of “closed-short” exchanges in the entire sample. This tight clustering signals that nearly six out of every 10 moves reliably elicit brief student responses, minimizing off-task or ambiguous talk. Coupled with the top ranking (1st), the statistic suggests that linguistic predictability functions as a calibrated scaffold which, by reducing processing load, channels student attention to accurate, fluent output and is rewarded by the judges. In contrast, teachers with lower rankings (e.g., C, E) have higher non-elicitation rates, suggesting that effective elicitation of student responses correlates with contest performance.

**Table 5:** Descriptive statistics of class question and answer combinations in the linguistic dimension.

**Teacher**	**Q-A combination**	**Cell range**	**Attractor state**	**Attractor value (%)**	**Dispersion Coefficient**	**Grid Visit Entropy**	**Ranking**
A	39	8	Closed-short	58.97	0.665	1.862	1
B	31	9	Non-elicitation	25.81	0.888	2.005	6
C	40	9	Non-elicitation	47.50	0.771	1.933	10
D	30	7	Closed-short	53.33	0.690	1.622	4
E	37	7	Non-elicitation	40.54	0.728	1.557	7
F	23	5	Closed-short	52.17	0.698	1.545	3
G	34	8	Closed-short	44.12	0.766	1.711	8
H	18	5	Closed-short	38.89	0.784	1.437	2
I	34	8	Closed-short	44.12	0.736	1.688	5
J	29	7	Closed-short	41.38	0.743	1.658	9
Sum	315	12^*^	\	\	\	\	\
Average	31.5	7.35	\	44.683	0.778	1.564	\

^*^This figure reports the total number of grid cells visited within the 16-cell interaction space across the full sample; duplicate visits are collapsed into single counts, providing a measure of collective coverage rather than an additive aggregation of individual ranges.

#### 5.2.2 TSI in the cognitive dimension

**Figure 4:**
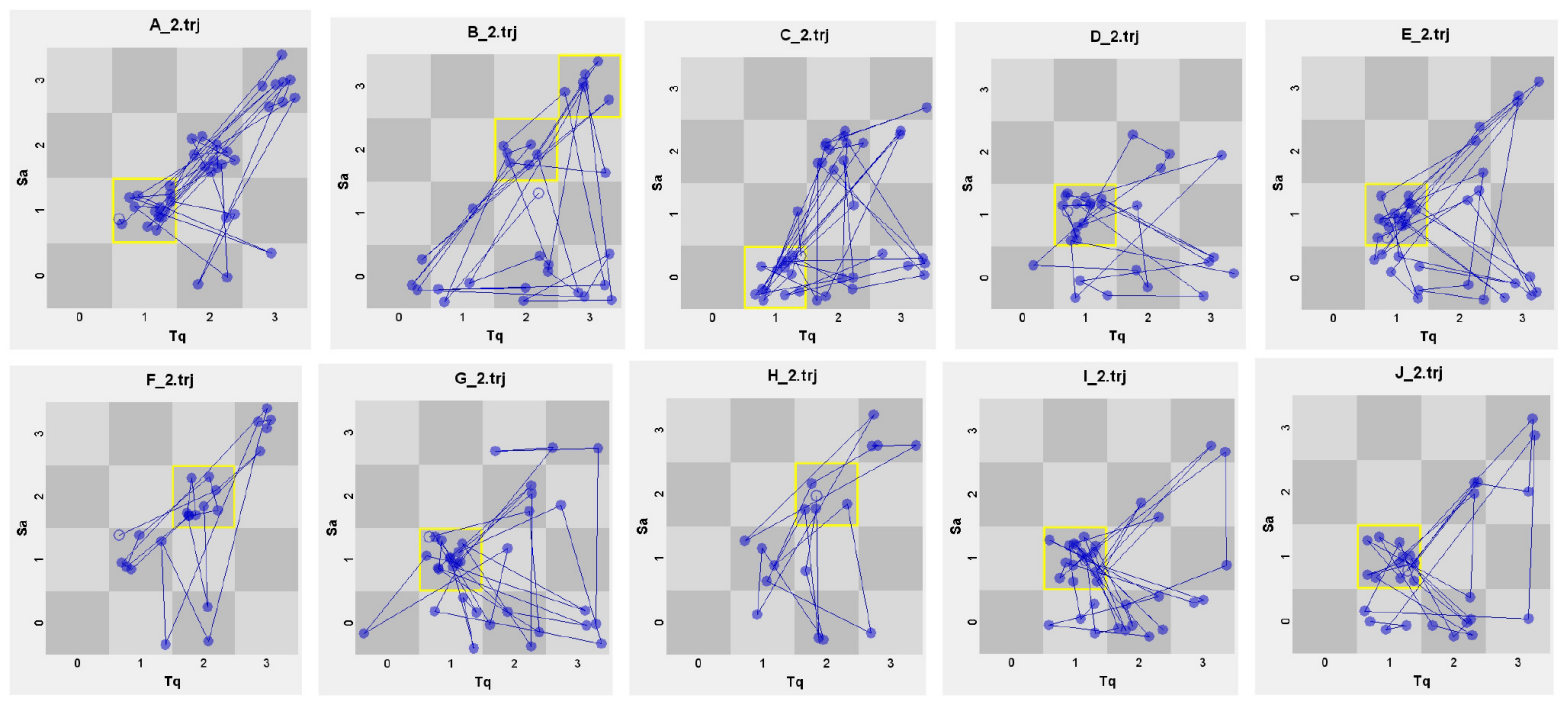
TSI of individual teachers in the cognitive dimension.

For the cognitive dimension, we parsed the 10 teachers' questioning trajectories in Figure 4 and Table 6. Sharp disparities exist in these interaction patterns, with attractor states varying a lot. Teacher B has the lowest attractor value (19.35%), highest dispersion coefficient (0.908) and highest grid visit entropy (2.045), indicating that her cognitive-level moves are the most fragmented and unpredictable of the cohort. Teacher A, in contrast, has the lowest dispersion coefficient (0.773) among all teachers, and although her attractor value (38.46%) is not the absolute highest, the tight clustering around the “low-low” state shows that her cognitive questions form a compact, repeatable routine with a low-level elicitation delivered with minimal variation. This controlled predictability, mirrored by her top ranking, suggests that cognitive consistency rather than sheer complexity is the dimension the judges reward.

**Table 6:** Descriptive statistics of class question and answer combinations in the functional dimension.

**Teacher**	**Q-A combination**	**Cell range**	**Attractor state**	**Attractor value (%)**	**Dispersion coefficient**	**Grid visit entropy**	**Ranking**
A	39	6	Low-low	38.46	0.773	1.568	1
B	31	9	Medium-medium/High-high	19.35	0.908	2.045	6
C	40	8	Low-N/A	35.00	0.817	1.769	10
D	30	8	Low-low	50.00	0.751	1.793	4
E	37	7	Low-low	40.54	0.813	1.822	7
F	23	5	Medium-medium	39.13	0.770	1.512	3
G	34	10	Low-low	38.24	0.851	2.013	8
H	18	7	Medium-medium	27.78	0.856	1.750	2
I	34	7	Low-low	47.06	0.758	1.625	5
J	29	7	Low-low	41.38	0.799	1.734	9
Sum	315	12	\	\	\	\	\
Average	31.5	7.35	\	37.694	0.778	1.732	\

### 5.3 Correlation analysis

#### 5.3.1 Relationship between teacher question and student answer

To further investigate the temporal interplay between Teacher-question (Tq) and Student-answer (Sa), we computed Spearman's correlations across two synchronized series, namely, ordinal-time (zero-lag) and lagged-time (Tq shifted one step forward), separately for the linguistic and cognitive dimensions (Table 7).

**Table 7:** Spearman's correlation analysis of teacher-student question and answer relationships.

**Teacher**	Ordinal Time QandA Series				Lagged Time QandA Series			
	Linguistic		Cognitive		Linguistic		Cognitive	
	ρ	***p***	ρ	***p***	ρ	***p***	ρ	***p***
A	0.474^**^	0.002	0.706^***^	<0.001	0.297	0.070	0.084	0.616
B	0.600^***^	<0.001	0.443^*^	0.012	0.375^*^	0.041	0.199	0.293
C	0.798^***^	<0.001	0.392^*^	0.012	0.141	0.390	0.281	0.083
D	0.573^***^	<0.001	−0.063	0.740	0.407^*^	0.028	−032	0.870
E	0.873^***^	<0.001	0.231	0.169	−350	0.839	−112	0.517
F	0.978^***^	<0.001	0.789^***^	<0.001	0.024	0.915	0.276	0.214
G	0.697^***^	<0.001	0.227	0.196	0.096	0.596	0.384^*^	0.028
H	0.979^***^	<0.001	0.559^*^	0.016	0.083	0.752	0.000	1.000
I	0.853^***^	<0.001	0.146	0.409	0.257	0.149	0.190	0.290
J	0.887^***^	<0.001	0.336	0.051	0.214	0.165	−093	0.638
Average	0.771		0.377		0.154		0.118	

^*^p < 0.05 (significant), ^**^p < 0.01 (highly significant), ^***^p < 0.001 (extremely significant). applied hereafter.

Ordinal-time series capture instantaneous contingency, i.e., how tightly Sa mirrors Tq complexity at the same discursive moment. Linguistically, all 10 teachers yield large positive coefficients (mean ρ = 0.771; min = 0.474^**^ for Teacher A, max = 0.979^***^ for Teacher H) with very high significance, evidencing a rigid turn-by-turn coupling: when a teacher raises (or lowers) linguistic demand, students almost immediately calibrate their reply to that level. Cognitively, the linkage relaxes (average ρ = 0.377); only five coefficients are significant (*p* < 0.05) and Teacher D registers a negative, non-significant ρ = −0.063, implying that higher-order prompts do not systematically evoke commensurately higher-order responses.

Lagged-time series examine contingent scaffolding, or whether the complexity of Tq at turn *t* + 1 is conditioned by Sa at turn *t*. Here correlations collapse: linguistic ρ averages.154 (only 2/10 significant), cognitive ρ averages.118 (1/10 significant). The strongest lagged effect appears for Teacher B in the linguistic dimension (ρ = 0.375^*^) and for Teacher G in the cognitive dimension (ρ = 0.384^*^); the remaining coefficients hover near zero. This pervasive absence of lagged significance suggests that award-winning teachers, despite their real-time synchrony with students, do not consistently modulate subsequent question complexity as a function of the preceding answer's sophistication.

#### 5.3.2 Relationship between linguistic and cognitive dimensions

To further explore the relationship between the cognitive and linguistic dimensions embodied in the TSI of award-winning teachers and their students, a Spearman's correlation analysis was conducted (Table 8). The analysis revealed that both dimensions exhibited statistically highly significant positive correlations with the target construct across both teacher questions and student answers, reflecting the inherent connection between language form and cognitive meaning in interactive communication. Notably, the correlation was exceptionally strong in student answers (ρ = 0.930^***^), indicating that students' linguistic expressions and cognitive engagement were nearly synchronized in response to teachers' guidance. In contrast, a moderately strong yet still highly significant correlation was observed in teacher questions (ρ = 0.391^***^) with 7 independent teachers' correlation significant.

**Table 8:** Spearman's correlation analysis of the relationship between linguistic and cognitive dimensions in TSI.

**Teacher**	Tq		Sa	
	ρ	***p***	ρ	***p***
A	0.792^***^	<0.001	0.846^***^	<0.001
B	0.504^**^	0.004	0.915^***^	<0.001
C	0.474^**^	0.002	0.953^***^	<0.001
D	0.573^***^	<0.001	0.919^***^	<0.001
E	0.252	0.132	0.895^***^	<0.001
F	0.717^***^	<0.001	0.896^***^	<0.001
G	0.327	0.059	0.948^***^	<0.001
H	0.562^*^	0.015	0.886^***^	<0.001
I	0.278	0.112	0.979^***^	<0.001
J	0.455^*^	0.013	0.982^***^	<0.001
All	0.391^***^	<0.001	0.930^***^	<0.001

^*^p < 0.05 (significant), ^**^p < 0.01 (highly significant), ^***^p < 0.001 (extremely significant).

## 6 Discussion

To investigate how global TSI patterns emerge from the local dynamics of individual educators, this study analyzed classroom interaction episodes involving 10 award-winning teachers. Drawing on the theoretical lens of CDST, SSGs were employed to capture and visualize complex dynamics inherent in these interactions at both the global level and local levels.

### 6.1 Global TSI patterns among award-winning teachers

Regarding RQ1, it is revealed that the dominant TSI pattern consists of simple questions paired with short answers, characterized by low cognitive demand, primarily involving factual recall rather than higher-order thinking skills such as analysis, evaluation, or creation (Anderson and Krathwohl, 2001). From the perspective of CDST, these dominant patterns constitute attractor states, stable configurations that occur far more frequently than by chance and substantially more often than most other possible interaction states (Hollenstein, 2013; Hiver and Larsen-Freeman, 2020). Consistent with previous research (Smit et al., 2022; Huang et al., 2025; Peng and Yu, 2025), this finding confirms the prevalence of low-complexity interactions between teachers and students, typically involving closed-ended questions and concise responses. This phenomenon stems from at least two interrelated factors. First, closed-ended questions allow teachers to quickly gauge student comprehension, ensuring smooth communication. This is particularly critical in contests, where interaction fluency and time management are key evaluation criteria (Hu, 2021). Second, this pattern may also stem from the somewhat performative nature of teaching particularly in contest settings (Alexander et al., 2005; Pineau, 2005): teachers often have the opportunity to prepare and rehearse general classroom interactions in advance, leading to a higher frequency of structured, low-cognitive-load exchanges that align with the expected performance norms (Long and Sato, 1983).

Based on the empirical findings, this study further elucidates the nuanced interplay between cognitive demands and linguistic performance in the EFL classroom. The observed misalignment between teacher-initiated higher-order thinking and student output resonates with the CDST perspective, underscoring the nonlinear relationship between pedagogical input and learning outcomes, as cognitive engagement does not automatically translate into linguistically sophisticated production due to constraints in learners' inter language systems (Larsen-Freeman and Cameron, 2008). Importantly, the near absence of cognitive “idling” suggests that teachers and students collectively sustain a high-cognitive-demand environment, actively calibrating the interaction toward deeper processing, a phenomenon consistent with the principles of collaborative meaning construction and scaffolded learning (Mercer and Dörnyei, 2020). Thus, while students may not consistently meet the intended linguistic-cognitive targets, the very act of engaging in and maintaining effortful reasoning reflects a dynamic, co-adaptive process wherein language and cognition interactively evolve through contextualized practice (Evans and Larsen-Freeman, 2020).

### 6.2 The emergence of global patterns from the local dynamics of individual teachers

Addressing RQ2, our analysis identifies variability and co-adaptation as the core CDST mechanisms driving the emergence of global interaction patterns from individual teachers' local dynamics (Larsen-Freeman and Cameron, 2008; de Bot et al., 2007a; Verspoor and Lowie, 2020). Consistent with CDST's anti-reductionist stance (Thelen and Smith, 1994; van Geert, 2008), global order arises from iterative local variability, an adaptive, generative force rather than random noise (Lowie and Verspoor, 2019; Verspoor and van Dijk, 2011). Besides, Our data confirm substantial cross-teacher variability in local dynamic indicators (e.g., cell range, attractor state strength), most notably in cognitive attractor states. This aligns with Qin et al. (2024). who found local syntactic variability preceded stable global complexity patterns in L2 writing. As (Thelen and Smith, 1994) emphasize, local variability facilitates functional self-organization: diverse cognitive attractor states across teachers converged on a global “adaptive cognitive scaffolding” pattern, one that no single teacher's local dynamics could predict. This echoes Kliesch and Pfenninger (2021), who linked learners' local linguistic variability to emergent global proficiency, confirming CDST's anti-reductionist stance (Larsen-Freeman, 2020).

A notable variability is top-ranking Teacher A's emergence as the sole participant who maintained zero non-elicitation turns, demonstrating exceptional consistency in both linguistic and cognitive alignment. When comparing these interaction patterns with contest rankings, a tentative conclusion emerges: the higher a teacher's ranking, the greater the depth and quality of teacher-student interaction. This finding aligns with prior research on outstanding language teachers, who typically facilitate more engaged and dynamic classroom exchanges than their peers (Moskowitz, 1976). In contrast to lower-ranking teachers, whose instructional practices aligned closely with structured teaching models (Long and Sato, 1983; Shi and Stickler, 2018), Teacher A's performance was recognized by judges for its integration of multiple interactional factors—consistent with Walsh (2011) framework of classroom interactional competence, which emphasizes the holistic evaluation of pedagogic discourse beyond rigid structural parameters.

However, co-adaptation, or mutual adjustments between system components (de Bot et al., 2007b), transforms local variability into coherent global patterns (Verspoor and van Dijk, 2011). Our analysis revealed three interconnected layers of local co-adaptation: between linguistic and cognitive dimensions, between teachers and students, and across temporal scales. These iterative loops amplify functional behaviors. Our divergent correlation findings (students: ρ = 0.930; teachers: ρ = 0.391) reflect CDST's differentiated component roles (van Geert and Steenbeek, 2005): teachers act as dynamic regulators, balancing dimensions flexibly, while students serve as adaptive responders who integrate them. This aligns with Yu and Lowie (2020) who linked local complexity-accuracy co-adaptation to stable global L2 speech patterns.

Intriguingly, teachers did not consistently modulate subsequent question complexity based on prior student responses, echoing Smit et al. (2022), and Huang et al. (2025). From CDST, this reflects context-sensitive, contingent local dynamics (de Bot, 2015) as global patterns arise from multiple local factors (immediate co-adaptation, attractor variability) rather than sole reliance on lagged adjustments, confirming emergence as a distributed process (Thibault, 2011).

Synthesizing the above analysis, we attribute the emergence of global TSI patterns to the following factors: (1) **Variability generation**: Individual teachers' local dynamics exhibit heterogeneous cognitive-linguistic interaction combinations, creating the “dynamic potential” for system change (Lowie and Verspoor, 2019); (2) **Co-adaptive binding**: Reciprocal adaptations between linguistic-cognitive dimensions, teachers and students, and immediate-lagged interactions filter and amplify functional local behaviors while suppressing non-functional ones (Huang et al., 2025); (3) **Systemic self-organization**: Over iterative interaction rounds, the aggregated co-adaptive behaviors self-organize into a coherent global pattern that possesses “emergent properties” not reducible to individual local dynamics (Byrne and Callaghan, 2014; Gui et al., 2021); (4) **Stabilization as attractor states**: The global pattern stabilizes as a dominant attractor state (Hollenstein, 2013), guiding subsequent local interactions while retaining flexibility to adapt to contextual changes (van Dijk et al., 2024). In our study, this explains why individual teachers' idiosyncratic local dynamics ultimately converged on a shared global pattern, proof that CDST's core tenets of variability, co-adaptation, and self-organization are applicable to classroom TSI research.

## 7 Conclusion

Guided by CDST and supplemented by SSGs to capture and visualize the intricate dynamics of TSI, this study explores how global interaction patterns emerge from the local adaptive behaviors of award-winning English teachers in China. The findings demonstrate that individual teachers' context-sensitive variability ultimately converges, via co-adaptation and systemic self-organization, into a shared global interaction pattern: the predominance of simple elicitation questions paired with concise student answers, a configuration characterized by relatively low cognitive challenge. Notably, Teacher A distinguishes herself as the sole participant who maintained zero non-elicitation turns, achieving exceptional consistency in both linguistic and cognitive alignment with the emergent pattern.

These findings have implications for advancing high-quality TSI in contemporary language education and refining the framework of China's national English teaching contests. First, for classroom instruction, the identified “simple-question-short-answer” pattern, while prevalent among award-winning teachers, highlights a critical area for pedagogical enhancement: integrating more cognitively demanding interactional moves (e.g., open-ended inquiries, scaffolded reasoning prompts) into routine TSI to foster deeper student engagement. Second, for teaching contest reform, the convergence of award-winning teachers toward a low-cognitive-demand interaction pattern signals a need to update contest evaluation criteria, shifting from surface-level interaction fluency to the quality of cognitive activation, and explicitly weighting practices that balance adaptability with intellectual challenge. Such revisions would align contest goals with national curriculum reforms prioritizing higher-order thinking skills in EFL education.

Despite its contributions, this study acknowledges some limitations. First, the sample size remains relatively modest, constraining the generalizability of the observed interaction patterns across different regional contexts and contest levels. Future research should expand the sample to include teachers across multiple contest tiers, enabling quantitative analysis of correlations between interactional features, teacher rankings, and student learning outcomes. Second, the reliance on verbal data, due to the inability to systematically code facial expressions, gestures, and other non-verbal cues from classroom videos, limits the comprehensiveness of the interactional analysis. Subsequent studies should adopt multimodal analytical frameworks, responding to Huang et al. (2024) call for holistic TSI research. Third, the exploration of causal mechanisms underlying the emergence of global patterns from local dynamics remains preliminary. Future work could employ longitudinal designs to trace how individual adaptive strategies co-evolve into stable interactional norms over multiple instructional cycles.

## Data Availability

The original contributions presented in the study are included in the article/supplementary material, further inquiries can be directed to the corresponding author.
